# Sevoflurane Inhibits Colon Cancer Progression by Inducing Cell Autophagy and Apoptosis through the ROS/Nrf2/P62 Pathway

**DOI:** 10.32604/or.2026.082954

**Published:** 2026-09-14

**Authors:** Xiangui Liu, Jianhua Liu, Qingbin Meng, Yongsheng Shao

**Affiliations:** 1 Department of Gastrointestinal Surgery, Traditional Chinese and Westemm Medicine Hospital of Wuhan, Tongji Medical College, Huazhong University of Science and Technology, Wuhan, China; 2 Department of Thyroid and Breast Surgery, Traditional Chinese and Westemm Medicine Hospital of Wuhan, Tongji Medical College, Huazhong University of Science and Technology, Wuhan, China

**Keywords:** Sevoflurane, ROS/Nrf2/p62, autophagy, apoptosis, colon cancer, colorectal cancer

## Abstract

**Objectives:** There is debate over the effect of sevoflurane (SEV) on different cancers. This study aims to explore SEV’s role in colon cancer (CC) progression. **Methods:** The CC cell lines were treated with SEV at concentrations of 1.7%, 3.4%, and 5.1%. Cell proliferation, apoptosis, migration, and invasion were assessed using Cell Counting Kit-8 (CCK-8), 5-ethynyl-2′-deoxyuridine (EdU) incorporation, colony formation assay, flow cytometry, Western blot, scratch assay, and Transwell assay. A xenograft tumor model was established to evaluate the effect of SEV *in vivo*. Expression levels of nuclear factor erythroid 2-related factor 2 (Nrf2), p62 (sequestosome-1), microtubule-associated protein 1 light chain 3 isoform II to I ratio (LC3-II/I), Beclin-1, Bcl-2-associated X protein (Bax), and B-cell lymphoma 2 (Bcl-2) were measured. N-acetyl-L-cysteine (NAC), a ROS scavenger, was applied to determine whether ROS-dependent Nrf2/p62 signaling participated in this process. **Results:** SEV inhibited CC cell proliferation, colony formation, migration, and invasion. At 5.1% SEV, cell proliferation was reduced by 54.0% (*p* < 0.001); colony formation decreased by 55.0% (*p* < 0.001); and wound closure rates dropped from 78.3% to 24.6% (*p* < 0.001) in HCT116 cells. Similar trends were observed in SW480 cells. Invaded cells were also significantly reduced in both cell lines (*p* < 0.001). SEV also induced CC apoptosis. In the xenograft model, treatment with SEV significantly suppressed tumor progression, as evidenced by reductions in both tumor volume and weight (*p* < 0.001). Mechanistically, SEV increased the LC3-II/I ratio and Beclin-1 expression, while decreasing Nrf2 and p62 expression both *in vitro* and *in vivo*. Moreover, NAC reversed the SEV-induced effects *in vitro*. **Conclusion:** SEV inhibits CC progression by inducing autophagy and apoptosis via the ROS/Nrf2/p62 pathway.

## 1 Introduction

Colorectal cancer (CRC) ranks as the third most commonly diagnosed malignancy worldwide, with approximately 1.9 million new cases and 904,000 deaths annually [1]. CRC is anatomically and biologically heterogeneous, comprising colon cancer (CC) and rectal cancer, among which CC accounts for about 70% of all CRC cases [2,3]. Despite advances in surgical resection and adjuvant chemotherapy [4,5,6], survival outcomes in advanced disease remain limited. Patients with distant metastases still face an unfavorable prognosis, with a 5-year survival rate of less than 15% [7]. Therefore, identifying modifiable perioperative factors that influence CC progression is of critical clinical importance.

Sevoflurane (SEV) is widely used in the perioperative setting for induction and maintenance of general anesthesia across various surgical procedures, including CC resections [8,9,10]. It possesses favorable pharmacological properties, such as a low blood-gas partition coefficient, which allows rapid induction of and recovery from anesthesia, making it particularly suitable for ambulatory and short-duration surgeries [11]. Nevertheless, its effects on cancer biology remain controversial [12]. Preclinical studies have reported contradictory findings: some suggest that SEV promotes malignant behaviors in certain cancer types, such as ovarian cancer [13], while others indicate an inhibitory role in hepatocellular carcinoma [14,15], bladder cancer [16], and lung cancer [17]. For CRC, SEV has been reported to markedly suppress tumor-cell growth and motility, reduce invasive potential, and promote apoptotic cell death [18,19,20]. However, systematic evaluations of its underlying regulatory mechanisms on CC cell lines remain limited.

Reactive oxygen species (ROS) and antioxidants serve as signaling molecules involved in the oxidative stress response. Under pathological conditions, excessive accumulation of ROS can disrupt cellular homeostasis, induce oxidative stress, and impair mitochondrial function [21,22]. During this process, oxidative stress often triggers autophagy [23]. Autophagy is regulated by ROS through intrinsic mechanisms that include both transcriptional and post-transcriptional regulation, involving multiple molecular signaling pathways such as the ROS/Nuclear factor erythroid 2-related factor 2 (Nrf2)/sequestosome-1 (p62) axis [24,25]. Nrf2 is a key regulator of antioxidant defense against oxidative and environmental stress. It is also involved in modulating autophagic activity by forming a reciprocal regulatory circuit with the autophagy adaptor p62. The Nrf2-p62 signaling loop is therefore closely involved in antioxidant defense and autophagy regulation, highlighting its potential relevance as a therapeutic target in cancer [26]. The ROS-mediated autophagic pathway has been reported to be intricately linked to cancer progression and development, playing an essential role in tumor cell activities, particularly in the context of cell death [27,28]. Interestingly, SEV is known to promote ROS accumulation in cancer cells [29,30,31]; however, whether SEV can exploit this ROS-driven pathway to regulate CC progression is still unknown.

Autophagy is a lysosome-dependent degradation process that recycles unwanted or damaged intracellular organelles and proteins. During autophagy, intracellular materials are engulfed and delivered for breakdown, after which the resulting metabolites are reutilized. This recycling mechanism supplies essential building blocks, supports metabolic demands and cell survival, and contributes to the maintenance of cellular equilibrium [32]. Autophagy is closely involved in maintaining malignant cell viability, making its modulation an attractive therapeutic approach in oncology [33,34]. In CRC, autophagy interacts closely with metastasis and epithelial-mesenchymal transition (EMT), acting as a potent regulator of cancer cell invasion. Moreover, this cell death mechanism can cooperate with apoptosis, another major form of programmed cell death, to inhibit or promote CRC cell apoptosis depending on the context [35]. Recent studies have demonstrated that anticancer therapies, such as the combination of curcumin and metformin [36], berberine [37], and artesunate [38], exert their antitumor effects by inducing both autophagy and apoptosis. Hence, the ability to target autophagy and/or apoptosis to trigger cancer cell death has important therapeutic implications for CRC.

Based on the above evidence, we hypothesized a clear mechanistic cascade. SEV treatment increases ROS generation in CC cells, and the elevated ROS level then acts as an upstream signal to modulate the Nrf2/p62 axis. The subsequent changes in this pathway lead to the induction of both autophagy and apoptosis. Collectively, these regulated cell-death mechanisms cooperate to restrain tumor growth and impair malignant cell proliferation, migration, and invasion. In this proposed model, ROS is the initiating trigger. Autophagy and apoptosis are not independent events but rather synergistic processes. Autophagy may initially serve as a stress-adaptive response that ultimately contributes to cell death, while apoptosis executes the final elimination of cancer cells. Accordingly, this study was designed to provide a quantitative evaluation of the proposed mechanistic cascade and to further clarify its potential role in the underlying biological process. We exposed CC cells to graded SEV doses to evaluate concentration-related biological responses, assessed tumor-associated behaviors and relevant protein changes, and further used N-acetyl-L-cysteine (NAC) to determine whether ROS/Nrf2/p62 signaling mediates the effects of SEV. Both *in vitro* experiments and an *in vivo* xenograft tumor model were performed to ensure consistency of the findings. Our results demonstrate that sevoflurane inhibits colon cancer progression by inducing autophagy and apoptosis through the ROS/Nrf2/p62 pathway.

## 2 Materials and Methods

### 2.1 Cell Culture and Treatment

The human normal colon epithelial cell line NCM460 (CL-0749) and the human CC cell lines HCT116 (CL-0096) and SW480 (CL-0223B) were purchased from Procell (Wuhan, Hubei, China), cultured in Dulbecco’s Modified Eagle’s Medium (DMEM) (L110KJ, BasalMedia, Shanghai, China) supplemented with 10% fetal bovine serum (FBS), 100 U/mL penicillin, and 100 μg/mL streptomycin in a 5% carbon dioxide (CO_2_) incubator at 37°C. Cells were detached using 0.25% trypsin and passaged. Before use, all cell lines underwent STR-based identity verification and were tested negative for mycoplasma contamination. Mycoplasma testing was performed monthly using the PlasmoTest™ kit (rep-pt1, InvivoGen, San Diego, CA, USA).

For SEV treatment, CC cells were placed in a sterile airtight container equipped with inlet and outlet connectors. The outlet was connected to a gas monitor (PM8060, Drager, Lübeck, Germany) to monitor the SEV concentration. Cells were exposed to SEV (mixed with 95% oxygen (O_2_) and 5% CO_2_, Sevorane, Abbott, Chicago, IL, USA) at concentrations of 1.7%, 3.4%, and 5.1% for 6 h. Control cells were treated with a 95% O_2_ + 5% CO_2_ gas mixture for 6 h. Following treatment, cells were detached and cultured for another 24 h. The three concentrations of SEV were selected based on previous literature [18,19,20] that used similar concentration ranges for *in vitro* studies of SEV. In addition, preliminary experiments were performed to confirm that these concentrations did not cause excessive cytotoxicity while effectively inducing the biological effects of interest.

CC cells were incubated with 5 mM NAC (1009005; Sigma-Aldrich, St. Louis, MO, USA) for 1 h before being subjected to hyperoxic conditions containing 95% O_2_/5% CO_2_ or 5.1% SEV exposure for 6 h, according to the protocol described above. Following treatment, cells were transferred from the container to the incubator and cultured for 24 h.

### 2.2 Cell Counting Kit-8 (CCK-8) Assay

Each well of a 96-well plate received 100 μL of cell culture medium containing 2 × 10^3^ cells. After overnight incubation allowed sufficient adherence, cells were treated with sevoflurane at 1.7%, 3.4%, or 5.1% for 6 h according to the assigned experimental protocol. After SEV exposure, cells were transferred out of the airtight chamber and cultured for another 24 h. Then, 10 μL of CCK-8 reagent (CK04, Dojindo, Tokyo, Japan) was dispensed into each well following the kit protocol, followed by incubation at 37°C for 2 h. The optical density in each well was recorded at 450 nm with a Synergy H1 plate reader (BioTek, Winooski, VT, USA).

### 2.3 5-Ethynyl-2′-Deoxyuridine (EdU)

Cells were seeded into 96-well plates at 6000 cells per well and incubated for overnight for attachment. After attachment, cells were exposed to sevoflurane (SEV) at concentrations of 1.7%, 3.4%, and 5.1% for 6 h under the conditions described above. After SEV exposure, the cells were transferred out of the airtight chamber and maintained in culture for another 24 h. Then, cells were incubated with 100 μL of diluted EdU solution for 2 h according to the EdU cell proliferation detection kit (C10310, RiboBio, Guangzhou, Guangdong, China). After EdU labeling, cells were fixed with 4% paraformaldehyde for 15 min, permeabilized with 0.5% Triton X-100 for 10 min, and then stained with DAPI (4′,6-diamidino-2-phenylindole) to visualize total nuclei. Images were captured under a fluorescence microscope (Leica, Wetzlar, Germany) in five randomly selected fields per well.

### 2.4 Colony Formation Assay

Cells were first exposed to SEV at concentrations of 1.7%, 3.4%, and 5.1% for 6 h under the conditions described above. Immediately after SEV exposure, cells were trypsinized, counted, and seeded into 6-well plates at 1000 cells per well. The cells were then cultured at 37°C with 5% CO_2_ for 14 days without further SEV treatment. Thereafter, cells were gently washed twice with phosphate-buffered saline (PBS; C0221A, Beyotime, Shanghai, China), fixed in methanol (Y037896, Beyotime) for 30 min, and stained with 0.1% crystal violet (Y268090, Beyotime). Colonies containing more than 50 cells were counted manually under an Olympus microscope (Tokyo, Japan). Clonogenic capacity was determined by dividing the counted colony number by the initial seeding cell number and multiplying by 100%. Relative changes were expressed after normalization against the control group.

### 2.5 Flow Cytometry for Apoptosis

For apoptosis detection, cells were seeded and allowed to attach overnight, then exposed to SEV at concentrations of 1.7%, 3.4%, and 5.1% for 6 h under the conditions described above. Following SEV treatment, cells were removed from the sealed container and incubated for an additional 24 h. After this recovery period, both the culture medium containing floating (apoptotic/dead) cells and the trypsinized adherent cells were collected and pooled together prior to centrifugation. Cells were detached using EDTA-free trypsin (0.25% trypsin, C0205, Beyotime) to avoid false-positive Annexin V staining. After centrifugation, cells were resuspended in 100 μL of 1× Binding Buffer. According to the Annexin V-fluorescein isothiocyanate (FITC) Apoptosis Detection Kit (C1062, Beyotime), cells were incubated with 5 μL of Annexin V-FITC and 10 μL of propidium iodide (PI). After a 10-min incubation at room temperature, cells were placed on ice and resuspended with 400 μL 1× Binding Buffer, followed by immediate apoptosis detection using a FACScan^®^ flow cytometer (BD Biosciences, Franklin Lakes, NJ, USA).

### 2.6 Scratch (Wound Healing) Assay

Cells were seeded into 6-well plates at a density of 6 × 10^5^ cells per well. When 90% confluence was reached, the cells were exposed to SEV at concentrations of 1.7%, 3.4%, and 5.1% for 6 h under the conditions described above. After SEV exposure, cells were taken out of the airtight chamber and cultured for another 24 h. After removal of the culture medium, a linear wound was generated with a 200 μL pipette tip. Non-adherent cells and cellular debris were removed by two gentle PBS rinses (C0221A, Beyotime), after which fresh FBS-free DMEM (L110KJ, BasalMedia) was supplied. The wound area was photographed at baseline and 24 h later using an Olympus light microscope. The wound area was measured using ImageJ software (version 1.53, National Institutes of Health, Bethesda, MD, USA). The wound healing rate was calculated using the formula: (wound area at 0 h − wound area at 24 h)/wound area at 0 h × 100%.

### 2.7 Transwell Invasion Assay

Cells underwent a 6-h SEV treatment using the previously specified experimental settings. After SEV exposure, the cells were taken out of the airtight chamber and cultured for another 24 h. Following recovery, cells were detached with trypsin, enumerated, and suspended in medium without serum. Matrigel (356234, BD Biosciences) diluted in culture medium was added to the upper chamber of the Transwell insert (Corning, 3422, New York, NY, USA) at 40 μL per well. Cells (5 × 10^4^/mL) were seeded into the upper chamber (200 μL per well), while the lower chamber contained medium supplemented with 10% FBS (FBS500, BasalMedia). Cells were cultured for 24 h and then immersed in 4% paraformaldehyde (P0099, Beyotime) for 20 min. Subsequently, 0.1% crystal violet was applied for 10 min, and the stained cells were visualized using an Olympus microscope (Tokyo, Japan). Invaded cells were quantified by capturing five randomly selected microscopic areas from each insert at 200×, followed by blinded manual enumeration. The average number of invaded cells per field was calculated and used for statistical analysis.

### 2.8 ROS Level Detection

After cell seeding and overnight attachment, cells were exposed to SEV at concentrations of 1.7%, 3.4%, and 5.1% for 6 h under the conditions described above, followed by a 24-h recovery incubation. After the recovery period, the culture medium containing floating (dead or dying) cells was collected, and the remaining adherent cells were detached using EDTA-free trypsin (C0205, Beyotime). The floating and detached cells were pooled, washed twice with PBS, and then resuspended in serum-free medium to obtain a single-cell suspension. Cells were subsequently loaded with DCFH-DA probe (10 μmol/L; E004, Nanjing Jiancheng Bioengineering Institute, Nanjing, Jiangsu, China) and maintained at 37°C for 20 min under light-protected conditions. Signal acquisition was performed with a FACScan^®^ flow cytometer (BD Biosciences, Cat. No. 342973; Franklin Lakes, NJ, USA) under fluorescence detection conditions of 485 nm excitation and 535 nm emission. A total of 10,000 events were acquired per sample, and the mean fluorescence intensity was analyzed using CellQuest software (BD Biosciences).

### 2.9 Western Blot

Total protein from tissues and cells was extracted using radio-immunoprecipitation assay (RIPA) buffer (P0013B, Beyotime). Protein concentrations were determined using the bicinchoninic acid (BCA) protein quantification kit (P0012, Beyotime). SDS-PAGE was conducted by applying 50 μg total protein to each gel lane. Following electrophoretic separation, protein samples were electroblotted onto polyvinylidene fluoride (PVDF) membranes (Merck Millipore, IPVH00010, Burlington, MA, USA). The membranes were blocked with 5% non-fat dry milk for 2 h and then incubated overnight at 4°C with primary antibody dilutions: Nrf2 (1:1000, 16396-1-AP, Proteintech), p62 (1:5000; 18420-1-AP, Proteintech), Bax (1:1000; #5023, Cell Signaling Technology, Danvers, MA, USA), Bcl-2 (1:1000, #3498, Cell Signaling Technology), cleaved caspase-3 (1:1000, 19677-1-AP, Proteintech), Beclin-1 (1:1000; #3495, Cell Signaling Technology), LC3 (1:1000; 14600-1-AP, Proteintech), and β-actin (1:5000; ab8227, Abcam). After washing, the membranes were incubated with the corresponding HRP-conjugated secondary antibody (1:5000; ab205718/ab205719, Abcam) for 1 h at room temperature. Protein bands were visualized using enhanced chemiluminescence (ECL) substrate (1705061, Bio-Rad, Hercules, CA, USA) and analyzed with ImageJ software (version 1.53, National Institutes of Health, Bethesda, MD, USA).

### 2.10 Immunofluorescence Staining

Cells were fixed with 4% paraformaldehyde for 15 min, blocked with 10% donkey serum (Solarbio, SL050, Beijing, China) for 1 h, and then incubated overnight at 4°C with the primary antibody against LC3 (microtubule-associated protein 1 light chain 3; 1:500; 14600-1-AP, Proteintech, Rosemont, IL, USA). After being rewarmed at 37°C for 35 min, the cells were exposed to a fluorophore-labeled secondary antibody (1:500; A-11034, Invitrogen, Waltham, MA, USA) for 2 h at room temperature, counterstained with DAPI (4′,6-diamidino-2-phenylindole; C1006, Beyotime) for 15 min, and photographed under a laser confocal microscope (Leica, Wetzlar, Germany).

### 2.11 In Vivo Assay

All animal procedures were performed in accordance with the ARRIVE guidelines 2.0 [39]. Ten 4-week-old BALB/C nude mice (Hunan SJA Laboratory Animal Co., Ltd., Hunan, China) were housed in a specific pathogen-free (SPF) facility under a 12-h light/dark cycle with ad libitum access to food and water. Mice were randomly divided into two groups of five mice each. HCT116 cells collected from control or 5.1% SEV-treated groups were resuspended at a density of 5 × 10^6^ cells/mL. The cell suspension (0.1 mL per mouse) was injected subcutaneously into the axillae of the mice. Tumor volume was measured every 7 days using the formula (L × W^2^)/2, where L is the longest diameter, and W is the shortest diameter (units: mm^3^). At the experimental endpoint (21 days after cell inoculation), all mice were euthanized by CO_2_ asphyxiation followed by cervical dislocation. The grafted tumors were completely excised, weighed (units: g), and photographed. This experiment was approved and reviewed by the Institutional Animal Care and Use Committee of Traditional Chinese and Westemm Medicine Hospital of Wuhan (Approval No. WIVA04202204).

Tumor specimens were preserved in 4% paraformaldehyde, processed through graded dehydration, embedded in paraffin blocks, and cut into 4-μm-thick sections for subsequent analysis. Paraffin-embedded tissue slides were dewaxed, passed through graded alcohols for hydration, treated with H_2_O_2_ to quench endogenous peroxidase activity, and followed by microwave-assisted epitope retrieval. The sections were incubated with 10% goat serum (SL038, Solarbio) for 30 min to minimize background staining caused by nonspecific antibody interactions. They were then incubated overnight at 4°C with primary antibodies: cleaved caspase-3 (1:200; 19677-1-AP, Proteintech), Nrf2 (1:200; 16396-1-AP, Proteintech), p62 (1:200; 18420-1-AP, Proteintech), and LC3 (1:200; 14600-1-AP, Proteintech), and 4-Hydroxynonenal (4-HNE; 68538-1-Ig, Proteintech). After rewarming for 60 min at room temperature, sections were incubated with a horseradish peroxidase (HRP)-conjugated secondary antibody (RGAR011 or RGAM011, Proteintech) for 30 min. Following diaminobenzidine (DAB) color development (P0202, Beyotime), sections were counterstained with hematoxylin (Y269827, Beyotime), sealed with neutral resin, and observed under a light microscope (Olympus, Tokyo, Japan).

### 2.12 Statistical Analysis

Statistical analyses were performed with GraphPad Prism 9 (GraphPad Software, San Diego, CA, USA). Continuous variables are presented as mean ± SD. Two-group differences were analyzed with an unpaired Student’s *t*-test. For datasets containing more than two groups, statistical differences were evaluated by one-way ANOVA, with post hoc pairwise testing conducted using Tukey’s method. Statistical significance was defined at a threshold of *p* < 0.05.

## 3 Results

### 3.1 SEV Inhibits CC Cell Proliferation, Migration and Invasion

**Figure 1:**
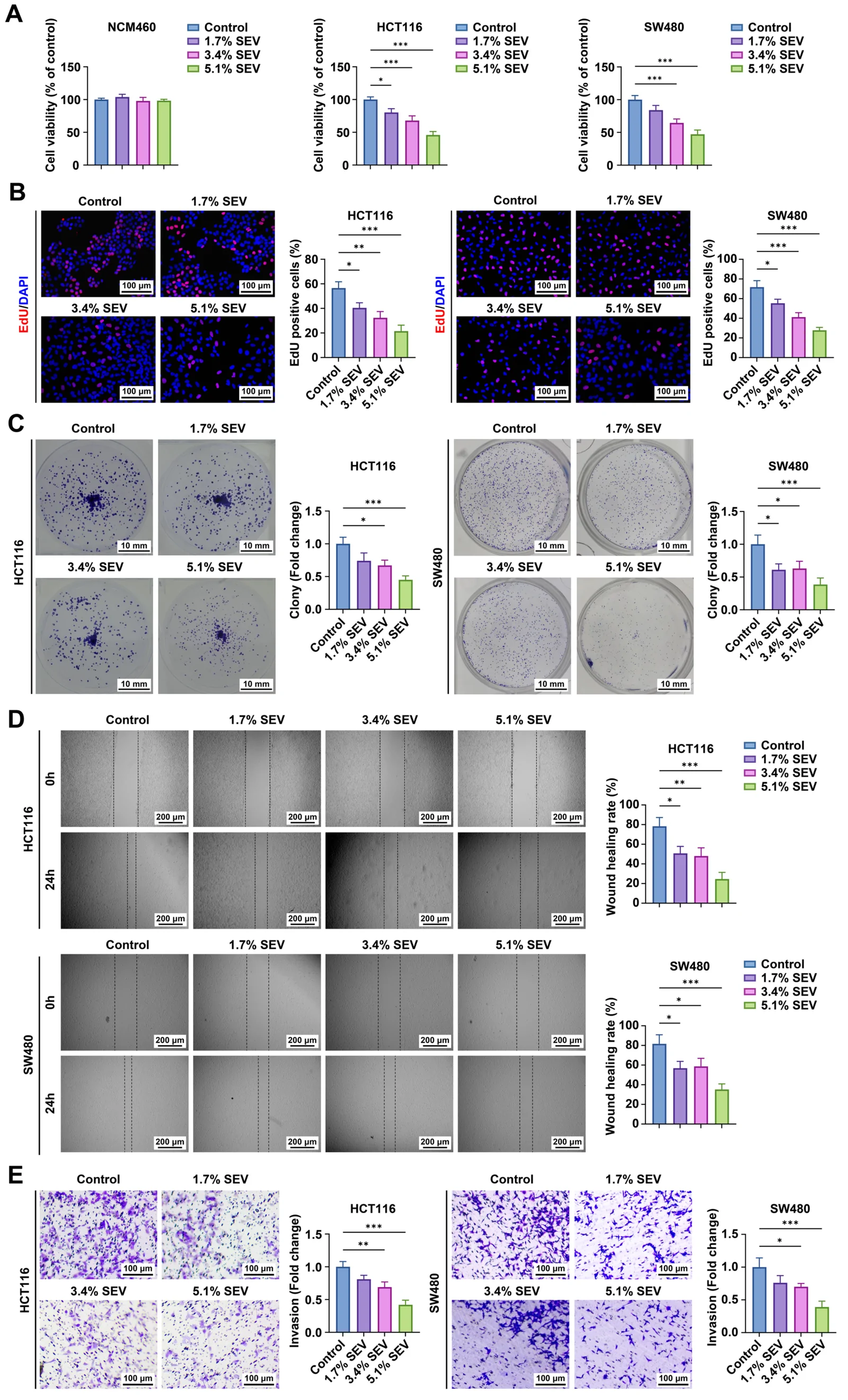
**Sevoflurane (SEV) inhibits colon cancer (CC) cell proliferation, migration and invasion.** (**A**) Cell viability of NCM460, HCT116, and SW480 cells treated with SEV was assessed using the Cell Counting Kit-8 (CCK-8) assay. (**B**) The proliferation of HCT116 and SW480 cells was evaluated by 5-ethynyl-2′-deoxyuridine (EdU) assay. Scale bar = 100 μm. (**C**) The colony formation ability of HCT116 and SW480 cells was determined by colony formation assay. Scale bar = 10 mm. (**D**) The migration capacity of HCT116 and SW480 cells was analyzed using a wound-healing assay. Scale bar = 200 μm. (**E**) The invasion capacity of HCT116 and SW480 cells was examined via Transwell invasion assay. Scale bar = 100 μm. Data are presented as the mean ± standard deviation (SD) of three independent experiments. *p < 0.05, **p < 0.01, ***p < 0.001.

The effects of SEV at concentrations of 1.7%, 3.4%, and 5.1% on the proliferation, migration, and invasion of CC cell lines were examined. The CCK-8 assay revealed that SEV inhibited cell viability in both HCT116 and SW480 cells, with the most pronounced inhibition observed at 5.1% SEV (Fig. 1A). EdU staining further confirmed that SEV inhibited cell proliferation in a dose-dependent manner, with the lowest proportion of EdU-labeled cells observed in the high-dose SEV group (Fig. 1B). Similarly, colony formation assays demonstrated that SEV decreased the clonogenic ability of both cell lines (Fig. 1C). Cell migration and invasion were assessed using scratch wound healing and Transwell assays. SEV exposure significantly weakened cell motility and invasiveness, with the most pronounced effect observed at 5.1% (Fig. 1D,E).

### 3.2 SEV Induces Apoptosis and Autophagy in Colon Cancer Cells

**Figure 2:**
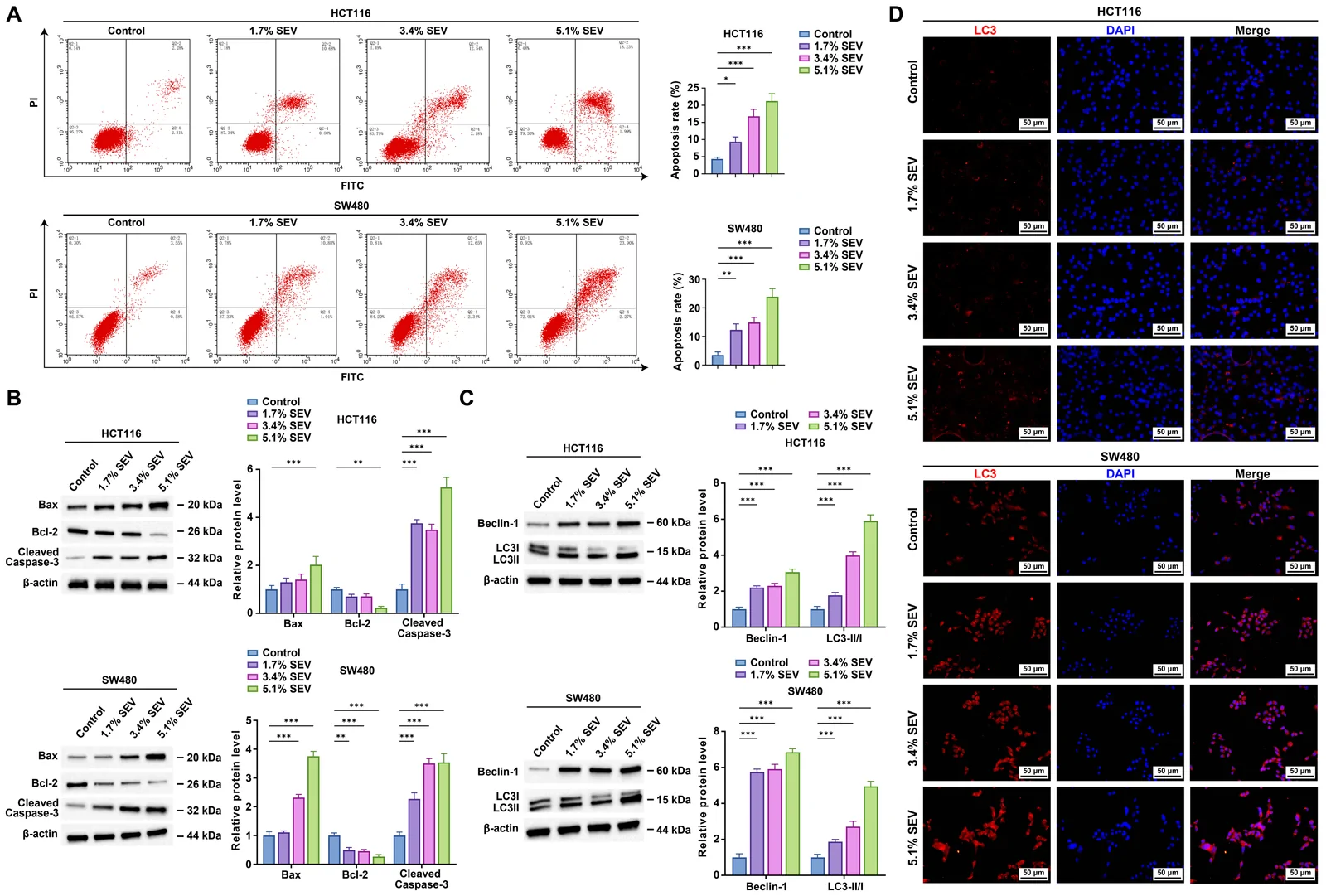
**Sevoflurane (SEV) promotes programmed cell death and autophagic activity in colon cancer (CC) cells.** (**A**) Flow cytometry was used to detect the effect of SEV on apoptosis in HCT116 and SW480 cells. (**B**) Western blotting was used to detect Bcl-2-associated X protein (Bax), B-cell lymphoma 2 (Bcl-2), and cleaved caspase-3 levels in SEV-treated HCT116 and SW480 cells. (**C**) Western blot analysis of the effect of SEV on the protein expression of Beclin-1 and microtubule-associated protein 1 light chain 3 (LC3) in HCT116 and SW480 cells. (**D**) Immunofluorescence staining was used to analyze the effect of SEV on LC3 protein expression in HCT116 and SW480 cells. Scale bars = 50 μm. Data are presented as the mean ± SD of three independent experiments. *p < 0.05, **p < 0.01, ***p < 0.001.

Flow cytometry analysis indicated that SEV increased the apoptosis rate in both HCT116 and SW480 cells, with the most pronounced increase observed at 5.1% SEV (Fig. 2A). Western blot further showed that SEV up-regulated the expression of the pro-apoptotic proteins Bax and cleaved caspase-3, while down-regulating the anti-apoptotic protein Bcl-2, and these changes were most evident at the 5.1% concentration (Fig. 2B). Regarding autophagy, Western blot results demonstrated that SEV significantly induced Beclin-1, a key protein required for autophagosome initiation, and also increased the LC3-II/LC3-I ratio (Fig. 2C). Immunofluorescence staining for LC3 provided additional evidence that SEV enhanced LC3 expression in both cell lines (Fig. 2D).

### 3.3 SEV Modulates the ROS/Nrf2/P62 Pathway in CC Cells

**Figure 3:**
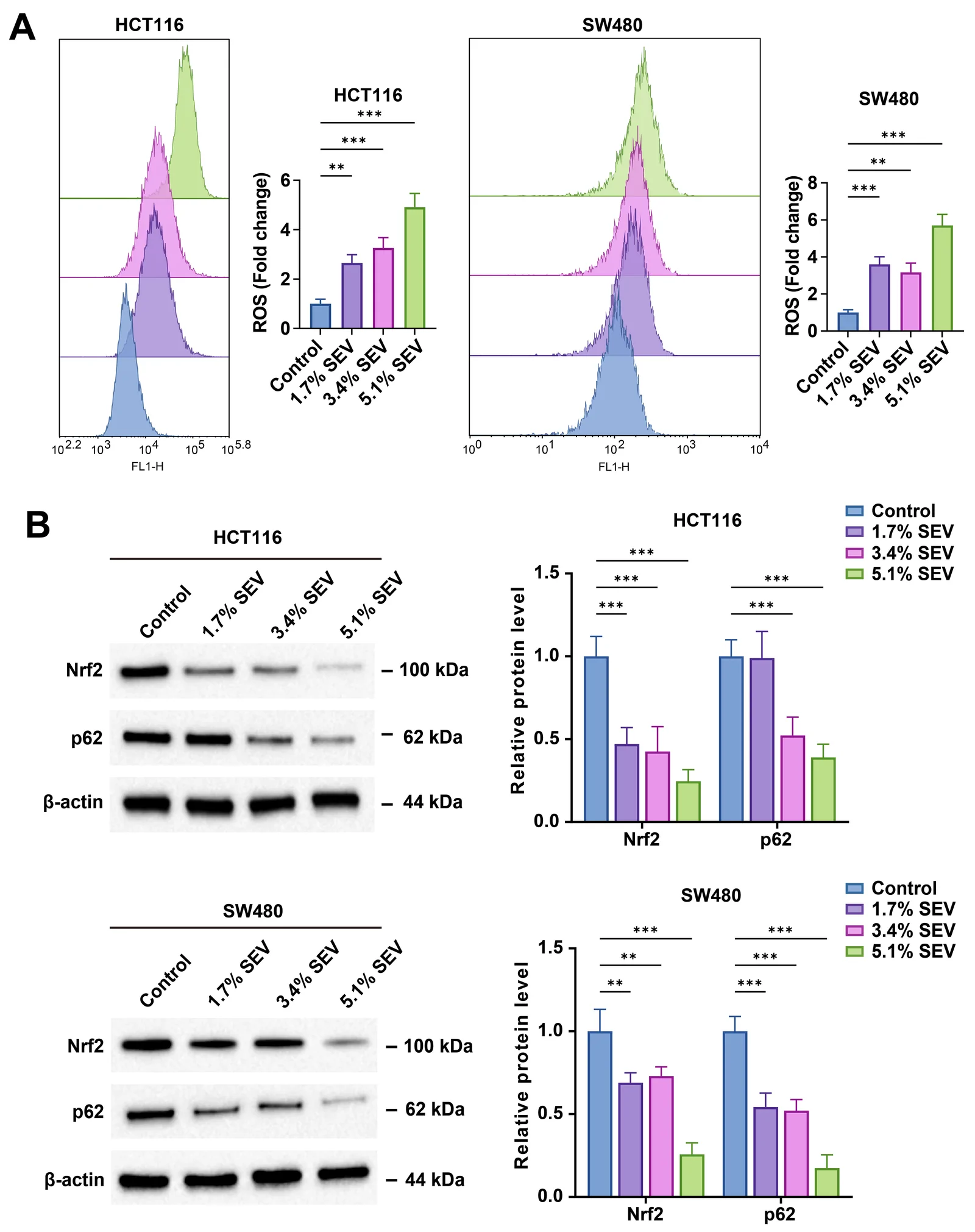
**Sevoflurane (SEV) modulates the reactive oxygen species (ROS)/nuclear factor erythroid 2-related factor 2 (Nrf2)/sequestosome-1 (p62) pathway in colon cancer (CC) cells.** (**A**) Intracellular ROS levels in HCT116 and SW480 cells following SEV treatment were evaluated by flow cytometry. (**B**) Nrf2 and p62 protein levels in HCT116 and SW480 cells following SEV treatment were evaluated by Western blotting. Data are presented as the mean ± SD of three independent experiments. **p < 0.01, ***p < 0.001.

Flow cytometry showed that SEV triggered an increase in ROS levels in both HCT116 and SW480 cells, with the most pronounced elevation observed at the 5.1% concentration (Fig. 3A). Moreover, western blot analysis revealed that SEV reduced the protein levels of Nrf2 and p62, and the reduction was strongest at the highest concentration (Fig. 3B).

### 3.4 NAC Counteracts SEV-Regulated Autophagy and Apoptotic Signaling in CC Cells

**Figure 4:**
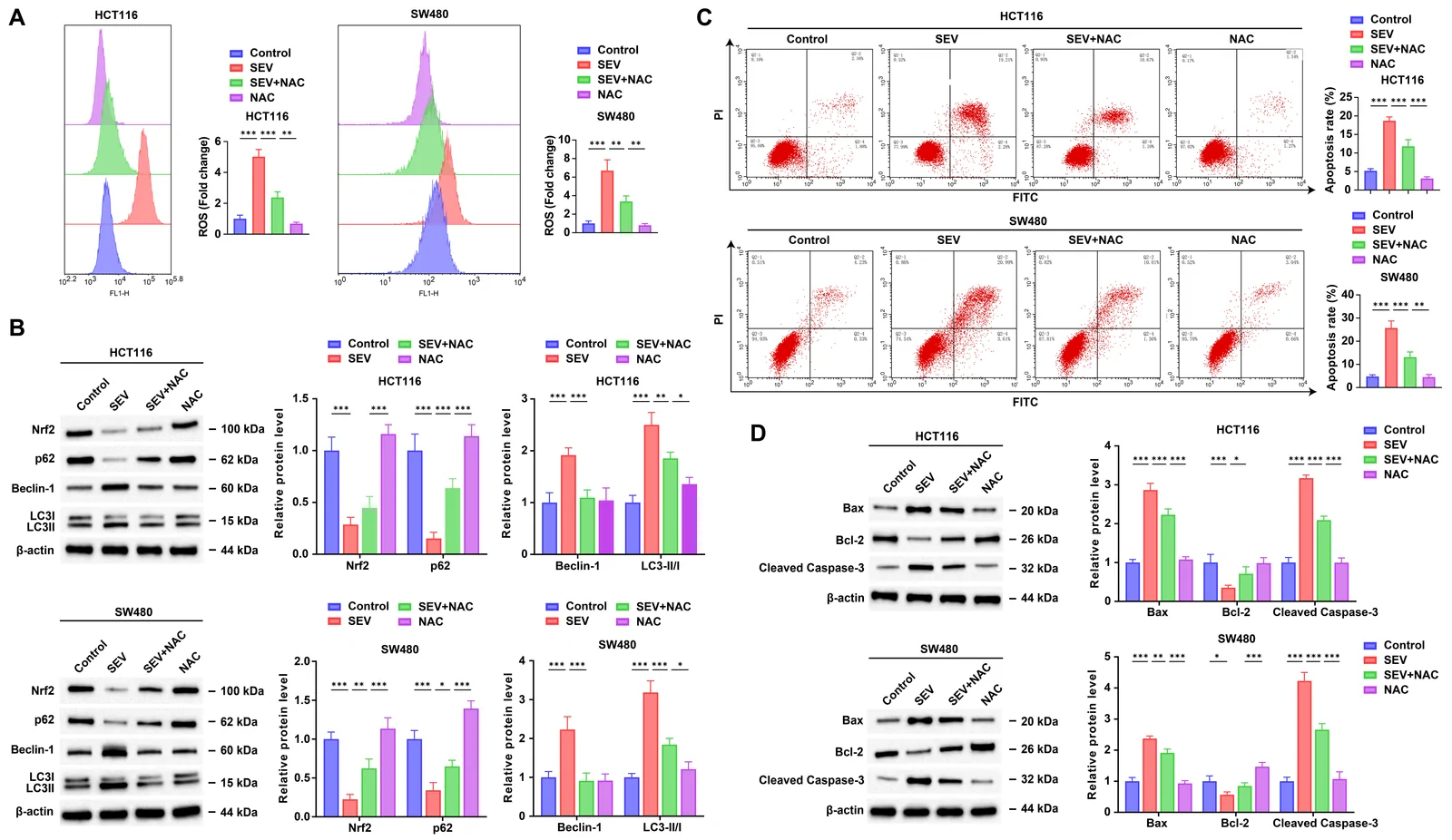
**N-acetyl-L-cysteine (NAC) reverses the Sevoflurane (SEV)-induced effects on autophagy and apoptosis in colon cancer (CC) cells.** (**A**) Intracellular ROS levels in CC cells treated with SEV, with or without NAC pretreatment, were evaluated by flow cytometric analysis. (**B**) Protein levels of Nrf2, p62, Beclin-1, and LC3 in CC cells after SEV exposure, with or without NAC preconditioning, were evaluated by western blotting. (**C**) Flow cytometry was used to detect the effect of SEV (with or without NAC pretreatment) on apoptosis in CC cells. (**D**) Western blot analysis of the effect of SEV (with or without NAC pretreatment) on Bax, Bcl-2, and cleaved caspase-3 protein expression. Values are expressed as the mean ± standard deviation (SD) from three independently repeated experiments. Statistical significance is indicated as follows: *p < 0.05, **p < 0.01, ***p < 0.001.

To determine whether SEV promotes autophagic activity and apoptotic responses in CC cells through ROS/Nrf2/p62 signaling, cells were pre-incubated with the ROS scavenger NAC prior to SEV exposure. Flow cytometry confirmed that NAC effectively attenuated the SEV-induced ROS accumulation (Fig. 4A). Western blot analysis demonstrated that NAC treatment effectively reversed the SEV-induced downregulation of Nrf2 and p62, as well as the SEV-induced upregulation of Beclin-1 and the LC3-II/LC3-I ratio (Fig. 4B). Flow cytometry further demonstrated that NAC pretreatment significantly decreased the apoptosis rate that had been elevated by SEV (Fig. 4C). Consistently, western blot analysis of apoptosis-related proteins showed that NAC partially reversed the SEV-induced changes, namely the downregulation of Bcl-2 and the upregulation of Bax and cleaved caspase-3 (Fig. 4D).

### 3.5 SEV Inhibits CC Progression In Vivo

**Figure 5:**
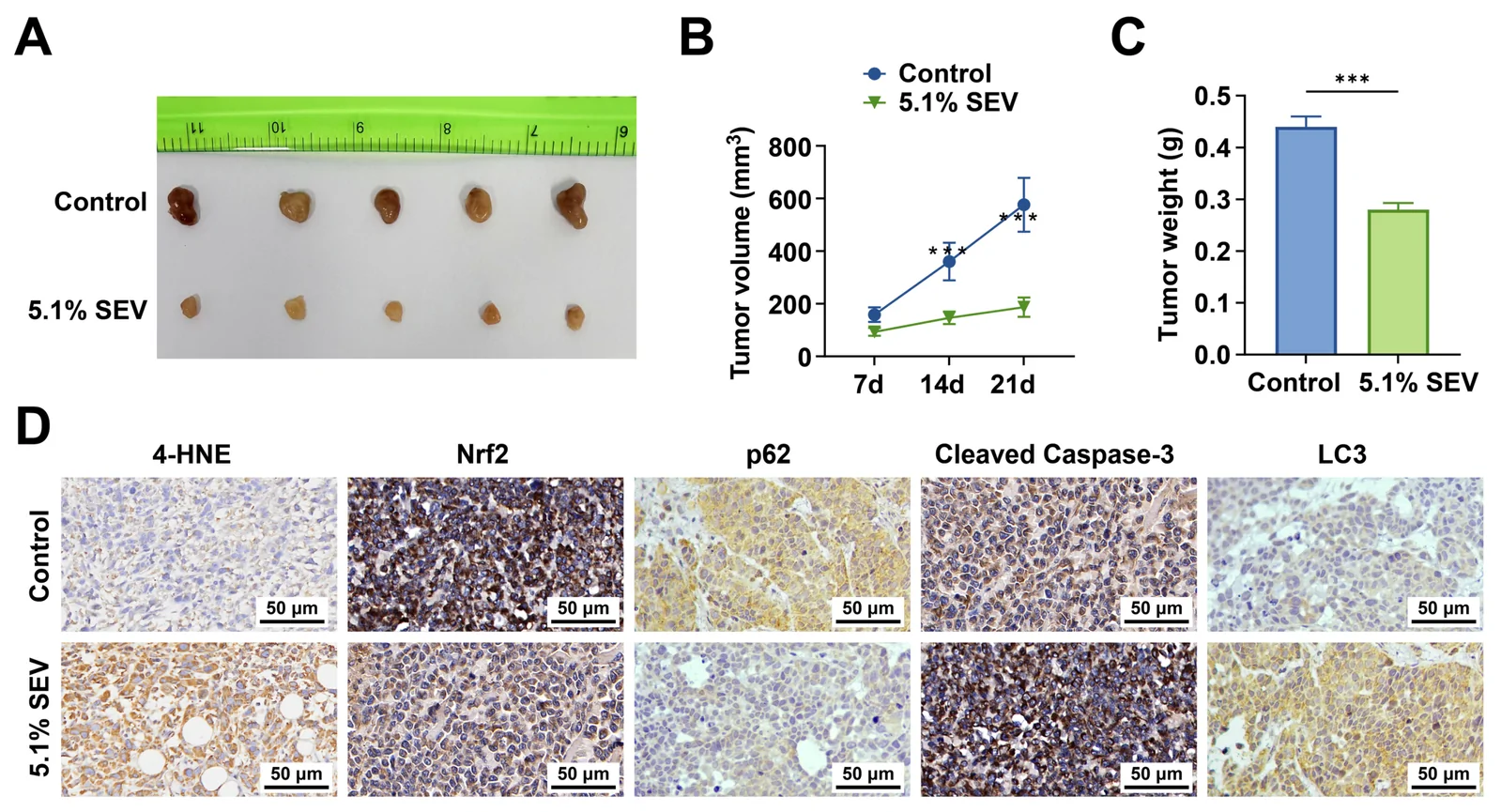
**Sevoflurane (SEV) inhibits colon cancer (CC) progression *in vivo*.** (**A**) Representative images of subcutaneous xenograft tumors from each group. (**B**) Tumor volume growth curves of the xenograft tumors over time. (**C**) Statistical analysis of the xenograft tumor weights. (**D**) Immunohistochemical (IHC) analysis of 4-hydroxynonenal (4-HNE), Nrf2, p62, Cleaved Caspase-3, and LC3 protein expression in the xenograft tumor tissues. Scale bar = 50 μm. Data are presented as the mean ± SD (*n* = 5 per group). ***p < 0.001.

In a xenograft mouse model, administration of 5.1% SEV markedly inhibited tumor growth, as evidenced by decreases in both tumor size and mass relative to the control group, demonstrating the *in vivo* anti-CC effect of SEV (Fig. 5A–C). More importantly, immunohistochemistry analysis of the transplanted tumor tissues revealed that the 5.1% SEV treatment group exhibited decreased expression of Nrf2 and p62, while the expression levels of 4-HNE, cleaved caspase-3 and LC3 were markedly increased (Fig. 5D). These changes in Nrf2, p62, cleaved caspase-3, and LC3 were consistent with the *in vitro* observations, while the increase in 4-HNE further indicated enhanced lipid peroxidation in tumor tissues.

## 4 Discussion

There is increasing interest in the potential of SEV to inhibit CC progression through multiple mechanisms. For instance, SEV has been shown to suppress CC cell malignancy by modulating circ-PI4KA [40]. Additionally, previous studies have shown that SEV can suppress CRC cell growth and metastatic potential, reverse EMT-related changes, and promote apoptotic cell death [41,42,43]. This result is in line with earlier studies. We observed that SEV suppressed the malignant phenotypes of both HCT116 and SW480 CC cells, while enhancing apoptosis and autophagy in a dose-dependent fashion.

However, the effects of SEV on cancer biology have been reported to vary across different tumor types, concentrations, and experimental conditions. Even within the same pathological context of CC, previous studies have found that exposure to 1% SEV for 6 h could enhance the proliferative capacity of CC cells (HCT116 and HT29) through potassium (ATP) channels [44]. A previous *in vitro* study reported that exposure to 1% or 2.5% sevoflurane produced no obvious changes in cell-cycle profiles or apoptotic levels in SW480 colon cancer cells [45]. Furthermore, an *in vivo* study showed that under non-surgical conditions, SEV promoted the growth of colon cancer (Caco-2) tumors [46]. These discrepancies may be attributed to differences in cell type-specific signaling pathways, baseline ROS levels, antioxidant capacity, and the duration and concentration of SEV exposure. In our study, we used clinically relevant concentrations (1.7%, 3.4%, and 5.1%) and observed consistent anti-proliferative and pro-apoptotic effects in two different CC cell lines (HCT116 and SW480). The consistency across these two lines, which have distinct genetic backgrounds (HCT116: KRAS mutant, wild-type p53; SW480: KRAS mutant, p53 mutant), suggests that the anti-CC effect of SEV is not highly dependent on p53 status.

Next, the induction of autophagy by SEV in these cells led us to explore the mechanistic insights into its regulation of the ROS/Nrf2/p62 pathway. ROS facilitate lipid peroxidation and generate products such as 4-HNE, which play important roles in various cell death mechanisms, including apoptosis and autophagy [47]. Consistent with previous reports on ROS-mediated signaling in colon cancer [48,49], our results showed that SEV increased ROS production in CC cells in a dose-dependent manner, which was likely linked to SEV-induced autophagy and apoptosis. Transcriptional regulation of autophagy by ROS occurs mainly in the nucleus, where elevated ROS activate Nrf2. Oxidative stimulation activates Nrf2, which interacts with ARE motifs within the p62 promoter region, thereby enhancing p62 transcription. p62 then facilitates Nrf2 nuclear translocation by promoting the selective autophagic degradation of Keap1 (the negative regulator of Nrf2), forming a positive feedback loop that amplifies cellular protection [26,50]. Our finding that SEV induced ROS accumulation and downregulated Nrf2 and p62 aligns with studies showing that apatinib exerts anticancer effects through the same mechanism [51,52].

Notably, Nrf2 can have a dual role in tumors. While transient Nrf2 activation protects normal cells from carcinogenesis, constitutive or prolonged Nrf2 activation in cancer cells promotes chemoresistance and tumor survival [53,54]. In the present study, SEV treatment led to reduced Nrf2 expression, which likely contributed to increased ROS levels and subsequent cell death. This suggests that SEV may overcome the protective function of Nrf2 in CC cells, shifting the balance toward pro-death outcomes. Regarding the interplay between autophagy and apoptosis, our data indicate that SEV induces both processes simultaneously. In our experimental setting, autophagy appeared to act in a cooperative rather than antagonistic manner with apoptosis, as blocking ROS with NAC suppressed both autophagic and apoptotic markers. This cooperation may be context-dependent, and further studies using genetic inhibition of autophagy (e.g., ATG5 or Beclin-1 knockdown) would help clarify whether autophagy directly contributes to cell death or primarily serves as a stress-adaptive response that precedes apoptosis.

Our findings raise the possibility that SEV, as a commonly used inhaled anesthetic, may have beneficial effects on CC outcomes when used during perioperative period. If these results are confirmed in clinical studies, SEV might be considered not only as an anesthetic but also as an adjunct anti-tumor strategy for patients undergoing CC resection. However, it is important to note that our study used supraphysiological concentrations (up to 5.1%) and short exposure durations (6 h *in vitro*, single exposure *in vivo*), whereas clinical anesthesia typically involves lower concentrations (around 1-2 MAC) and repeated or prolonged exposure in some cases. Therefore, extrapolation to clinical practice should be made with caution. Future well-designed prospective studies are needed to evaluate the impact of SEV anesthesia on long-term oncological outcomes, such as recurrence-free survival and overall survival, in CC patients.

We acknowledge several limitations in this study. First, using additional cell lines with diverse genetic backgrounds would strengthen the generalizability of our conclusions. Second, although our pharmacological data with NAC support the involvement of the ROS/Nrf2/p62 pathway, genetic interventions such as Nrf2 manipulation are needed to offer more definitive proof for causality establishment. Third, the small sample size in the animal study (*n* = 5 per group) limits statistical power and increases the risk of type II errors. Furthermore, due to resource constraints, we could not perform *in vivo* pathway blockade experiments, measure ROS directly in tumor tissues, or apply additional methods to confirm autophagy and apoptosis. Fourth, our animal model used SEV pretreatment of cells before inoculation, which does not reflect the clinical scenario where SEV exposure occurs after tumor establishment. This design cannot address the effects of SEV on the tumor microenvironment, immune system, or drug metabolism. A post-inoculation exposure model would be more clinically relevant. Finally, we did not assess long-term animal survival or tumor recurrence, both of which are important for translational relevance. Future investigations should address the aforementioned limitations by incorporating multiple CC cell lines, performing genetic rescue experiments, adopting more clinically relevant animal models, and assessing long-term oncological outcomes.

## 5 Conclusions

This study demonstrates that SEV suppresses CC progression by inducing autophagy and apoptosis. Mechanistically, SEV increases ROS accumulation, which in turn downregulates the Nrf2/p62 pathway, leading to enhanced autophagic flux and apoptotic cell death. These effects were consistently observed in two CC cell lines (HCT116 and SW480) and were further validated in a xenograft mouse model. Our findings highlight the potential of SEV not only as an anesthetic but also as an adjunct anti-cancer strategy for CC, although future studies using more clinically relevant animal models and genetic interventions are warranted to strengthen the causal evidence.

## Data Availability

Data is available from the corresponding author on request.

## References

[1] Bray F , Laversanne M , Sung H , Ferlay J , Siegel RL , Soerjomataram I , et al. Global cancer statistics 2022: GLOBOCAN estimates of incidence and mortality worldwide for 36 cancers in 185 countries. CA Cancer J Clin. 2024; 74( 3): 229– 63. doi:10.3322/caac.21834. 38572751

[2] Siegel RL , Miller KD , Goding Sauer A , Fedewa SA , Butterly LF , Anderson JC , et al. Colorectal cancer statistics, 2020. CA Cancer J Clin. 2020; 70( 3): 145– 64. doi:10.3322/caac.21601. 32133645

[3] Baidoun F , Elshiwy K , Elkeraie Y , Merjaneh Z , Khoudari G , Sarmini MT , et al. Colorectal cancer epidemiology: Recent trends and impact on outcomes. Curr Drug Targets. 2021; 22( 9): 998– 1009. doi:10.2174/1389450121999201117115717. 33208072

[4] Biller LH , Schrag D . Diagnosis and treatment of metastatic colorectal cancer: A review. JAMA. 2021; 325( 7): 669. doi:10.1001/jama.2021.0106. 33591350

[5] Fabregas JC , Ramnaraign B , George TJ . Clinical updates for colon cancer care in 2022. Clin Colorectal Cancer. 2022; 21( 3): 198– 203. doi:10.1016/j.clcc.2022.05.006. 35729033

[6] Khan FA , Albalawi R , Pottoo FH . Trends in targeted delivery of nanomaterials in colon cancer diagnosis and treatment. Med Res Rev. 2022; 42( 1): 227– 58. doi:10.1002/med.21809. 33891325

[7] Emile SH , Horesh N , Wignakumar A , Boutros M , Wexner SD . Advanced T stage and nodal disease are independently associated with worse cancer-specific survival in stage IV colorectal cancer: A SEER-based survival analysis. Eur J Surg Oncol. 2026; 52( 2): 111315. doi:10.1016/j.ejso.2025.111315. 41418575

[8] Wu L , Li Y , Si J . Effects of sevoflurane and propofol on postoperative nausea and vomiting in patients with colorectal cancer placed under general anesthesia: A systematic review and meta-analysis. J Gastrointest Oncol. 2022; 13( 6): 2963– 72. doi:10.21037/jgo-22-783. PMC9830366 36636047

[9] Shen ZY , Wang J , Shen XP , Xv BQ , Shen DP . Comparative analysis of the effects of propofol and sevoflurane on coagulation and immune system function in patients undergoing radical surgery for colon cancer. Int J Gen Med. 2025; 18: 4457– 67. doi:10.2147/IJGM.S509276. PMC12360391 40831606

[10] Taavo M , Frithiof R , Enlund M , Franzén S . Acute kidney injury after propofol or sevoflurane anaesthesia for colorectal cancer surgery: A secondary analysis. Anaesthesia. 2026; 81( 5): 741– 3. doi:10.1111/anae.70198. PMC13065943 41804572

[11] Sun M , Xie Z , Zhang J , Leng Y . Mechanistic insight into sevoflurane-associated developmental neurotoxicity. Cell Biol Toxicol. 2022; 38( 6): 927– 43. doi:10.1007/s10565-021-09677-y. PMC9750936 34766256

[12] Hirai T , Konishi Y , Mizuno S , Rui Z , Sun Y , Nishiwaki K . Differential effects of sevoflurane on the growth and apoptosis of human cancer cell lines. J Anesth. 2020; 34( 1): 47– 57. doi:10.1007/s00540-019-02701-w. 31667585

[13] Hu C , Wang B , Liu Z , Chen Q , Ishikawa M , Lin H , et al. Sevoflurane but not propofol enhances ovarian cancer cell biology through regulating cellular metabolic and signaling mechanisms. Cell Biol Toxicol. 2023; 39( 4): 1395– 411. doi:10.1007/s10565-022-09766-6. PMC10425485 36207479

[14] Zhu X , Peng C , Peng Z , Chang R , Guo Q . Sevoflurane inhibits metastasis in hepatocellular carcinoma by inhibiting miR-665-induced activation of the ERK/MMP pathway. Cell Transplant. 2022; 31: 9636897221104447. doi:10.1177/09636897221104447. PMC9201366 35699095

[15] Sun N , Gong J , Zhang W , Yang X , Liu J . Sevoflurane suppresses hepatocellular carcinoma cell progression via circ_0001649/miR-19a-3p/SGTB axis. Histol Histopathol. 2023; 38( 5): 537– 47. doi:10.14670/HH-18-484. 35747942

[16] Lin L , Kong L , Dong X , Ma S . Sevoflurane suppresses growth and metastasis of bladder cancer cells via inducing ferroptosis and antitumor microenvironment through Akt/mTOR/SREBP1 signaling. Chem Biol Interact. 2025; 421: 111766. doi:10.1016/j.cbi.2025.111766. 41043699

[17] Zhou P , Yang L , Ma X , Li Q . Sevoflurane inhibits lung cancer development by promoting FUS1 transcription via downregulating IRF6. Carcinogenesis. 2024; 45( 8): 543– 55. doi:10.1093/carcin/bgae034. 38819072

[18] Wu T , Sun L , Wang C , Yu P , Cheng L , Chen Y . Sevoflurane suppresses the migration, invasion, and epithelial-mesenchymal transition of breast cancer cells through the miR-139-5p/ARF6 axis. J Surg Res. 2021; 258: 314– 23. doi:10.1016/j.jss.2020.08.051. 33317757

[19] Hu N , Duan JA , Yu Y , Li D , Chen J , Yan H . Sevoflurane inhibits the migration, invasion and induces apoptosis by regulating the expression of WNT1 via miR-637 in colorectal cancer. Anticancer Drugs. 2021; 32( 5): 537– 47. doi:10.1097/CAD.0000000000001061. 33735116

[20] Lu Y , Li L , Piao Z , Tan X , Su R . Sevoflurane and propofol co-affect the development of colorectal cancer by regulating TM2D1. Comb Chem High Throughput Screen. 2025; 28( 10): 1669– 78. doi:10.2174/0113862073281046240527165415. 38920064

[21] Nakamura H , Takada K . Reactive oxygen species in cancer: Current findings and future directions. Cancer Sci. 2021; 112( 10): 3945– 52. doi:10.1111/cas.15068. PMC8486193 34286881

[22] Sarmiento-Salinas FL , Perez-Gonzalez A , Acosta-Casique A , Ix-Ballote A , Diaz A , Treviño S , et al. Reactive oxygen species: Role in carcinogenesis, cancer cell signaling and tumor progression. Life Sci. 2021; 284: 119942. doi:10.1016/j.lfs.2021.119942. 34506835

[23] Su LJ , Zhang JH , Gomez H , Murugan R , Hong X , Xu D , et al. Reactive oxygen species-induced lipid peroxidation in apoptosis, autophagy, and ferroptosis. Oxid Med Cell Longev. 2019; 2019: 5080843. doi:10.1155/2019/5080843. PMC6815535 31737171

[24] Li D , Ding Z , Du K , Ye X , Cheng S . Reactive oxygen species as a link between antioxidant pathways and autophagy. Oxid Med Cell Longev. 2021; 2021: 5583215. doi:10.1155/2021/5583215. PMC8324391 34336103

[25] Redza-Dutordoir M , Averill-Bates DA . Interactions between reactive oxygen species and autophagy: Special issue: Death mechanisms in cellular homeostasis. Biochim Biophys Acta Mol Cell Res. 2021; 1868( 8): 119041. doi:10.1016/J.BBAMCR.2021.119041. 33872672

[26] Shan C , Wang Y , Wang Y . The crosstalk between autophagy and Nrf2 signaling in cancer: From biology to clinical applications. Int J Biol Sci. 2024; 20( 15): 6181– 206. doi:10.7150/ijbs.103187. PMC11628323 39664581

[27] Gao L , Loveless J , Shay C , Teng Y . Targeting ROS-mediated crosstalk between autophagy and apoptosis in cancer. Adv Exp Med Biol. 2020; 1260: 1– 12. doi:10.1007/978-3-030-42667-5_1. 32304028

[28] Ju S , Singh MK , Han S , Ranbhise J , Ha J , Choe W , et al. Oxidative stress and cancer therapy: Controlling cancer cells using reactive oxygen species. Int J Mol Sci. 2024; 25( 22): 12387. doi:10.3390/ijms252212387. PMC11595237 39596452

[29] Han XC , Zhang YJ , Dong X , Xing QZ , Li KH , Zhang L . Sevoflurane modulates the cancer stem cell-like properties and mitochondrial membrane potential of glioma via Ca^2+^-dependent CaMKII/JNK cascade. Life Sci. 2020; 253: 117675. doi:10.1016/j.lfs.2020.117675. 32360621

[30] Peng X , Zhou Y . Sevoflurane inhibits the malignant behavior of nasopharyngeal carcinoma cells by regulating mitochondrial membrane potential. Gen Physiol Biophys. 2024; 43( 4): 291– 300. doi:10.4149/gpb_2024014. 38953572

[31] Wang H , Cheng G , Zhang S , Qu H , Zhao X , Yang A , et al. Sevoflurane: A dual modulator of miR-211-5p and mitochondrial apoptosis in glioma therapy. Mol Med Rep. 2025; 32( 1): 179. doi:10.3892/mmr.2025.13544. PMC12046963 40280112

[32] Hernandez GA , Perera RM . Autophagy in cancer cell remodeling and quality control. Mol Cell. 2022; 82( 8): 1514– 27. doi:10.1016/j.molcel.2022.03.023. PMC9119670 35452618

[33] Liu J , Wu Y , Meng S , Xu P , Li S , Li Y , et al. Selective autophagy in cancer: Mechanisms, therapeutic implications, and future perspectives. Mol Cancer. 2024; 23( 1): 22. doi:10.1186/s12943-024-01934-y. PMC10807193 38262996

[34] Niu X , You Q , Hou K , Tian Y , Wei P , Zhu Y , et al. Autophagy in cancer development, immune evasion, and drug resistance. Drug Resist Updat. 2025; 78: 101170. doi:10.1016/j.drup.2024.101170. 39603146

[35] Zhang Z , Zhao Y , Wang Y , Zhao Y , Guo J . Autophagy/ferroptosis in colorectal cancer: Carcinogenic view and nanoparticle-mediated cell death regulation. Environ Res. 2023; 238( Pt 2): 117006. doi:10.1016/j.envres.2023.117006. 37669735

[36] Chuang HY , Chan HW , Shih KC . Suppression of colorectal cancer growth: Interplay between curcumin and metformin through DMT1 downregulation and ROS-mediated pathways. BioFactors. 2025; 51( 1): e2137. doi:10.1002/biof.2137. PMC11681316 39607347

[37] Shen F , Zheng YS , Dong L , Cao Z , Cao J . Enhanced tumor suppression in colorectal cancer via berberine-loaded PEG-PLGA nanoparticles. Front Pharmacol. 2024; 15: 1500731. doi:10.3389/fphar.2024.1500731. PMC11563832 39555093

[38] Fan X , Yan Y , Li Y , Song Y , Li B . Anti-tumor mechanism of artesunate. Front Pharmacol. 2024; 15: 1483049. doi:10.3389/fphar.2024.1483049. PMC11549674 39525639

[39] Percie du Sert N , Hurst V , Ahluwalia A , Alam S , Avey MT , Baker M , et al. The ARRIVE guidelines 2.0: Updated guidelines for reporting animal research. Br J Pharmacol. 2020; 177( 16): 3617– 24. doi:10.1111/bph.15193. PMC7393194 32662519

[40] Sun S , Wang P , Ren L , Wang H , Zhan Y , Shan S . Sevoflurane suppresses colon cancer cell malignancy by regulating circ-PI4KA. Onco Targets Ther. 2021; 14: 3319– 33. doi:10.2147/OTT.S295552. PMC8144176 34045869

[41] Wang J , Li S , Zhang G , Han H . Sevoflurane inhibits malignant progression of colorectal cancer via hsa_circ_0000231-mediated miR-622. J Biol Res-Thessalon. 2021; 28( 1): 14. doi:10.1186/s40709-021-00145-6. PMC8237491 34183076

[42] Kang X , Li X , Li Y . Sevoflurane suppresses the proliferation, migration and invasion of colorectal cancer through regulating Circ_0000423/miR-525-5p/SGPP1 network. Cell Mol Bioeng. 2022; 15( 2): 219– 30. doi:10.1007/s12195-021-00717-5. PMC8938590 35401845

[43] Song W , Miao L , Zhang K , Liu Y , Lin J , Li J , et al. Sevoflurane suppresses colorectal cancer malignancy by modulating β-catenin ubiquitination degradation via circSKA3. Cell Signal. 2024; 114: 110987. doi:10.1016/j.cellsig.2023.110987. 38029946

[44] Sugimoto H , Kawaraguchi Y , Nomura Y , Nishiwada T , Uemura K , Furuya H , et al. Exposure to 1% sevoflurane for 6 h enhances proliferation of human colon cancer cells. Masui. 2015; 64( 4): 357– 61. 26419095

[45] Bundscherer AC , Ullrich V , Malsy M , Gruber MA , Graf BM , Brockhoff G , et al. Effects of volatile anesthetics on proliferation and viability of SW480 colon cancer cells *in vitro*. Anticancer Res. 2019; 39( 11): 6049– 55. doi:10.21873/anticanres.13811. 31704831

[46] Iwasaki M , Zhao H , Hu C , Saito J , Wu L , Sherwin A , et al. The differential cancer growth associated with anaesthetics in a cancer xenograft model of mice: Mechanisms and implications of postoperative cancer recurrence. Cell Biol Toxicol. 2023; 39( 4): 1561– 75. doi:10.1007/s10565-022-09747-9. PMC10425502 35953652

[47] Wang B , Wang Y , Zhang J , Hu C , Jiang J , Li Y , et al. ROS-induced lipid peroxidation modulates cell death outcome: Mechanisms behind apoptosis, autophagy, and ferroptosis. Arch Toxicol. 2023; 97( 6): 1439– 51. doi:10.1007/s00204-023-03476-6. 37127681

[48] Shen J , Dong J , Shao F , Zhao J , Gong L , Wang H , et al. Graphene oxide induces autophagy and apoptosis via the ROS-dependent AMPK/mTOR/ULK-1 pathway in colorectal cancer cells. Nanomedicine. 2022; 17( 9): 591– 605. doi:10.2217/nnm-2022-0030. 35394351

[49] Chen JS , Chiu SC , Huang SY , Chang SF , Liao KF . Isolinderalactone induces apoptosis, autophagy, cell cycle arrest and MAPK activation through ROS-mediated signaling in colorectal cancer cell lines. Int J Mol Sci. 2023; 24( 18): 14246. doi:10.3390/ijms241814246. PMC10532319 37762548

[50] Bartolini D , Dallaglio K , Torquato P , Piroddi M , Galli F . Nrf2-p62 autophagy pathway and its response to oxidative stress in hepatocellular carcinoma. Transl Res. 2018; 193: 54– 71. doi:10.1016/j.trsl.2017.11.007. 29274776

[51] Sun X , Li J , Li Y , Wang S , Li Q . Apatinib, a novel tyrosine kinase inhibitor, promotes ROS-dependent apoptosis and autophagy via the Nrf2/HO-1 pathway in ovarian cancer cells. Oxid Med Cell Longev. 2020; 2020: 3145182. doi:10.1155/2020/3145182. PMC7244982 32509141

[52] Xie C , Zhou X , Liang C , Li X , Ge M , Chen Y , et al. Apatinib triggers autophagic and apoptotic cell death via VEGFR2/STAT3/PD-L1 and ROS/Nrf2/p62 signaling in lung cancer. J Exp Clin Cancer Res. 2021; 40( 1): 266. doi:10.1186/s13046-021-02069-4. PMC8385858 34429133

[53] Panieri E , Pinho SA , Afonso GJM , Oliveira PJ , Cunha-Oliveira T , Saso L . NRF2 and mitochondrial function in cancer and cancer stem cells. Cells. 2022; 11( 15): 2401. doi:10.3390/cells11152401. PMC9367715 35954245

[54] Bae T , Hallis SP , Kwak MK . Hypoxia, oxidative stress, and the interplay of HIFs and NRF2 signaling in cancer. Exp Mol Med. 2024; 56( 3): 501– 14. doi:10.1038/s12276-024-01180-8. PMC10985007 38424190

